# Diversion

**Published:** 1880-10

**Authors:** 


### DIVERSION.

Dentists, especially those outside of large cities, are often unable to decide upon some interesting, entertaining, instructive, and profitable outside occupation, that shall divert the attention and effort from the fatigue and worry of daily practice.

Various resorts for this purpose have been made, some wise and some otherwise, some profitable and some expensive, some instructive, some the reverse.

Some have taken to horses. Well, a horse is a good thing if he does not run away with his owner. There are some, who in years past were good and successful practitioners, who are now simply hanging on to the ragged edge of the profession because of equine love.

Some have taken to sheep and generally they have had the wool pulled over their eyes. A dentist might manage two or three sheep, but when they flock they should be turned over to the shepherd.

Some greatly fancy the gun and dog business. Well, that may do, if taken in broken doses; it is not quite appropriate for a daily diet; once a moon is sufficient. It is hard work and doubtful whether the game is worth the powder.

Poultry is said to be a good and interesting diversion for the dentist. So it may be if eggs and pedigree does not receive more attention than office and patients.

Flowers and fruit are cultivated by some as a diversion, and often with profit. This is a delightful occupation, and one that under favorable circumstances, will afford great pleasure and benefit.

A favorite diversion, with some, is in company with the bees. The study of bees is interesting, facinating, instructive, and profitable. A close study of the habits of these little creatures always arouses interest, and generally enthusiasm.

Some very interesting and important physiological facts and processes can be studied, very satisfactorily, by forming a partnership with the bees.

Brothers, go in for the bees. You may not, at first, have a great fancy for them, but the more you become acquainted with them, the better you will like them.

A few—not many—take to whisky and its relations, for recreation and diversion. It is a good thing for diversion, but bad for recreation.

The earnest devotees become greatly diverted, and recreated backwards.

Professional Brothers, let whisky and all its kindred alone; but if you must form an alliance with it, then abandon the dental profession, and though you ruin yourself do not also disgrace your profession.

